# Readmission and hospital mortality after ICU discharge of critically ill cancer patients

**DOI:** 10.1371/journal.pone.0211240

**Published:** 2019-01-24

**Authors:** Byeong-Ho Jeong, Soo Jin Na, Dae-Sang Lee, Chi Ryang Chung, Gee Young Suh, Kyeongman Jeon

**Affiliations:** 1 Division of Pulmonary and Critical Care Medicine, Department of Medicine, Samsung Medical Center, Sungkyunkwan University School of Medicine, Seoul, Republic of Korea; 2 Department of Critical Care Medicine, Samsung Medical Center, Sungkyunkwan University School of Medicine, Seoul, Republic of Korea; University of Notre Dame Australia, AUSTRALIA

## Abstract

**Background:**

Intensive care unit (ICU) readmission is generally associated with increased hospital stays and increased mortality. However, there are limited data on ICU readmission in critically ill cancer patients.

**Method:**

We conducted a retrospective cohort study based on the prospective registry of all critically ill cancer patients admitted to the oncology medical ICU between January 2012 and December 2013. After excluding patients who were discharged to another hospital or decided to end-of-life care, we divided the enrolled patients into four groups according to the time period from ICU discharge to unexpected events (ICU readmission or ward death) as follows: no (without ICU readmission or death, n = 456), early (within 2 days, n = 42), intermediate (between 2 and 7 days, n = 64), and late event groups (after 7 days of index ICU discharge, n = 129). The independent risk factors associated with ICU readmission or unexpected death after ICU discharge were also analyzed using multinomial logistic regression model.

**Results:**

There were no differences in the reasons for ICU readmission across the groups. ICU mortality did not differ among the groups, but hospital mortality was significantly higher in the late event group than in the early event group. Mechanical ventilation during ICU stay, tachycardia, decreased mental status, and thrombocytopenia on the day of index ICU discharge increased the risk of early ICU readmission or unexpected ward death, while admission through the emergency room and sepsis and respiratory failure as the reasons for index ICU admission were associated with increased risk of late readmission or unexpected ward death. Interestingly, recent chemotherapy within 4 weeks before index ICU admission was inversely associated with the risk of late readmission or unexpected ward death.

**Conclusion:**

In critically ill cancer patients, patient characteristics predicting ICU readmission or unexpected ward death were different according to the time period between index ICU discharge and the events.

## Introduction

The development of diagnostic and therapeutic modalities of cancer and improved clinical outcomes have caused an increase in the inflow of critically ill cancer patients to the intensive care unit (ICU) [1]. Despite recent studies suggesting the benefits of intensive care in cancer patients, it is also true that they still have worse clinical outcomes than patients without cancer [2,3]. Efforts have been made to identify the risk factors for ICU admission and mortality in cancer patients to improve their outcomes and to reduce the economic burden; however, there is limited data on unplanned ICU readmission and unexpected ward death after index ICU discharge in cancer patients, which is known to be associated with increased in-hospital mortality, length of hospital stay, and medical cost [4,5].

Previous studies that evaluated ICU readmission and unexpected ward death after ICU discharge have been performed in a general population of critically ill patients, not specifically in cancer patients, and have defined ICU readmission and unexpected ward death as an event that occurred during the entire hospital stay period. However, it is not well known whether all subsequent readmissions and unexpected ward death after ICU discharge have the same characteristics, particularly in cancer patients, who generally have a longer hospital stay [6].

Therefore, this study was conducted to investigate the characteristics and clinical outcomes of cancer patients that were readmitted to the ICU or unexpectedly died on the ward after being discharged alive and to identify patient-related risk factors for predicting ICU readmission or unexpected ward death according to the time period between index ICU discharge and the events.

## Materials and methods

We conducted a retrospective cohort study based on the prospective registry of all critically ill patients with active cancer, defined as diagnosis of cancer, and any treatment for cancer within the previous six-month period or recurrent or metastatic cancer documented by medical record, admitted to the oncology medical ICU of the Samsung Comprehensive Cancer Center of Samsung Medical Center (a 1,979-bed tertiary referral hospital in Seoul, South Korea) between January 2012 and December 2013. The ICU has 14 beds and provides care for approximately 350 critically ill cancer patients per year [7]. The institutional review board of Samsung Medical Center approved this study and waived the requirement for informed consent because of the observational nature of the study.

### ICU admission, management, and discharge

In our hospital, intensivists are involved in decision-making for all admissions to the medical ICU. When the patients on the hospital ward show signs of clinical deterioration, their physician or nursing staff call the medical emergency team, including the intensivists, and then the medical emergency team decides whether to admit to the ICU [7]. Admission from an emergency room (ER) or other hospital is also determined through consultation with intensivists. Our ICU is a high-intensity ICU, and dedicated intensivists are responsible for day-to-day patient management. Multidisciplinary teams comprised of ICU physicians, bed-side nurses, nurse practitioners that specialize in respiratory care, clinical pharmacists, and nutritionists routinely assessed all patients admitted to the ICU. An ICU discharge was determined by discussion between the multidisciplinary team and the physician responsible for the patient at the general ward. There were no predefined criteria for admission and discharge, but decisions were made based on generally accepted guidelines [8]. Because our hospital does not have a step-down unit, all patients were discharged from the ICU to the general ward. Verbal and written handover was performed between medical staff members, and the ICU team did not routinely follow-up on the patients discharged from the ICU. Readmission to the ICU was decided in the same way as the index ICU admission.

### Study patients

All consecutive critically ill patients admitted to the oncology medical ICU were prospectively registered from admission. During the study period, a total of 1,125 active cancer patients were admitted to the ICU. We excluded patients who died during index ICU admission (n = 265), who were admitted for post-operative observation (n = 86), who were discharged to another hospital (n = 42), and who were decided not to readmit to the ICU with limitation of care decision by attending physician (n = 41); after exclusions, a total of 691 patients who were discharged alive to the general ward were included in this study.

Data on issues such as unplanned ICU readmission and unexpected death on the ward were collected until the patients were discharged from the ward. However, ICU readmission for scheduled procedures or post-operative observations were not counted as unplanned ICU readmission. If a patient was repeatedly admitted to the ICU during the same hospitalization, we collected the data for the first ICU admission as the index admission and the first ICU readmission as the end-point.

### Data collection

The following baseline characteristics were collected on each patient at index ICU admission: Demographic data and Eastern Cooperative Oncology Group [ECOG] performance status, comorbidities, status of malignancy, source of ICU admission, reason for ICU admission, Simplified Acute Physiology Score 3 [SAPS 3], and Sequential Organ Failure Assessment [SOFA] score. As this study included only patients with malignancies, other definitions associated with cancer status were defined as reported previously [9–11]. The extensiveness of the malignancy was classified according to the extent of the tumor and major organ involvement, as reported previously [9–12]. Extensive disease was defined as metastatic or locally extensive disease in patients with oncologic malignancies [13].

Based on the previous reports on unplanned ICU readmission [14–18], the primary outcome variables in this study were unplanned readmission to the ICU or unexpected death on the ward following ICU discharge. The following data and events were collected at index ICU discharge: organ support treatment during index ICU admission, ICU length of stay (LOS), SOFA score, vital signs, Glasgow coma scale (GCS), arterial blood gases, and laboratory findings. Finally, we collected data associated with status at the time of ICU readmission and final outcome at the time of hospital discharge.

### Statistical analyses

All data are presented as median and interquartile range (IQR) or as the number (percentage) of patients. The data were compared using the Kruskal-Wallis test for continuous variables and Pearson’s χ^2^ test or Fisher’s exact test for categorical variables. If there were multiple comparisons, we corrected the *P* value using the Bonferroni method.

We used multinomial logistic regression analysis to identify the independent variables associated with events (unplanned ICU readmission or unexpected death at the ward) depending on the time since index ICU discharge (defined as early, intermediate, and late event groups), with the no event group as the reference category. Multinomial logistic regression analysis was performed with backward stepwise selection with a *P* < 0.05 for entry of variables and *P* > 0.10 for removal of variables. Initial candidate variables were baseline characteristics at index ICU admission and data at index ICU discharge, except variables having missing data. Continuous variables were entered after converting to categorical variables to facilitate clinical interpretation. Results are presented as odd ratios (ORs) with 95% confidence intervals (CIs). All the tests were two-tailed, and a *P* value < 0.05 was considered significant. The data were analyzed using PASW Statistics 18 (SPSS Inc., Chicago, IL, USA).

## Results

**Fig 1:**
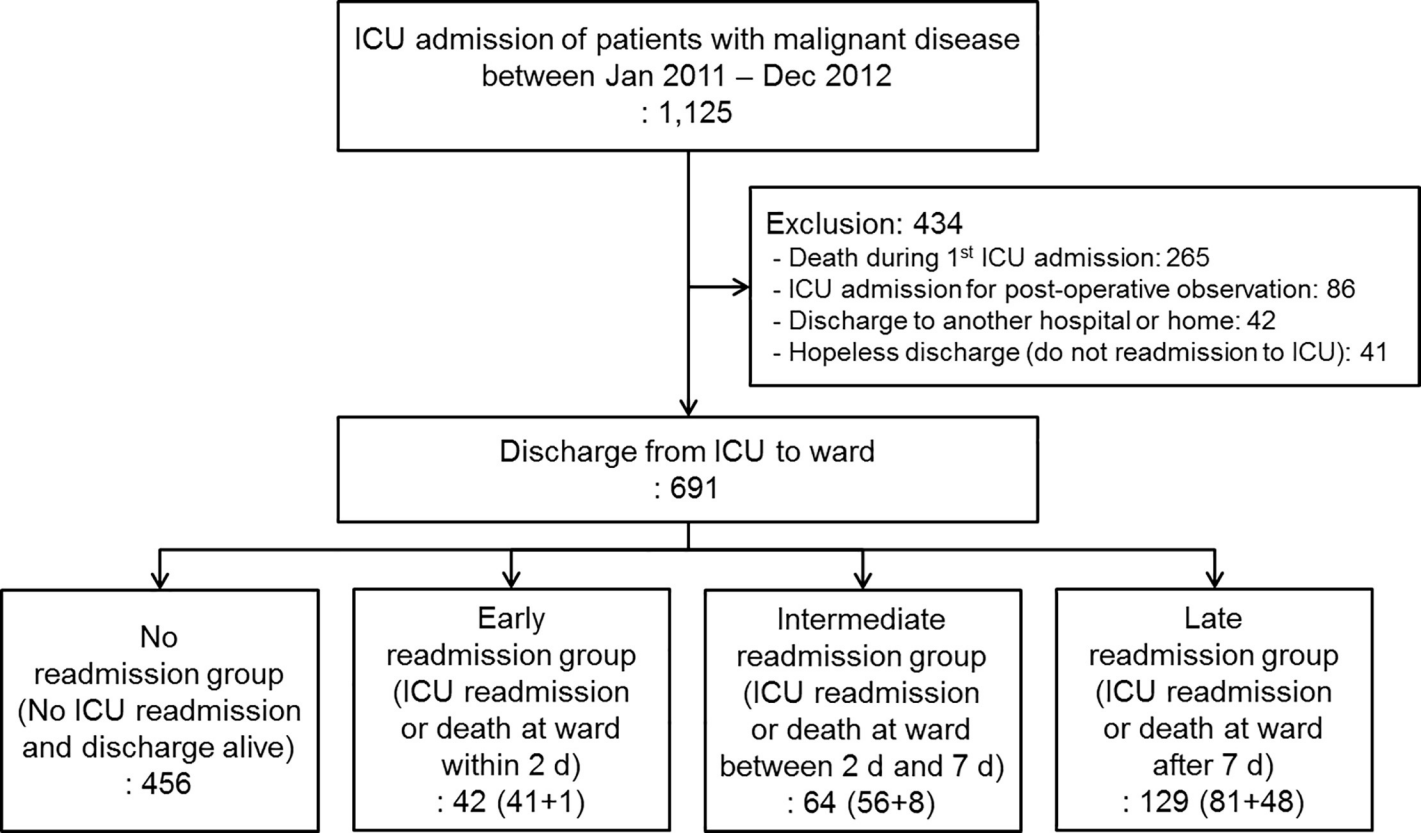
Patient distribution after being discharged alive from the intensive care unit (ICU). A total of 691 patients who were discharged alive from the medical ICU were divided into four groups: No event group, patients who were alive and discharged from hospital without ICU readmission; early event group, patients who were unexpectedly readmitted to the ICU or died on the ward within 2 days after index ICU discharge; intermediate event group, patients who were unexpectedly readmitted to the ICU or died on the ward between 2 and 7 days after index ICU discharge; late event group, patients who were unexpectedly readmitted to the ICU or died on the ward after 7 days of index ICU discharge.

Patient distribution after being discharged alive from the intensive care unit (ICU) is shown in Fig 1.

We divided the 691 enrolled patients into four groups as follows (Fig 1): (A) Patients who were discharged alive from the hospital without ICU readmission (no event group; n = 456, 66%), (B) patients who were unexpectedly readmitted to the ICU or died at the ward within 2 days after index ICU discharge (early event group; n = 42, 6%), (C) patients who were unexpectedly readmitted to the ICU or died at the ward between 2 and 7 days after index ICU discharge (intermediate event group; n = 64, 9%), and (D) patients who were unexpectedly readmitted to the ICU or died at the ward after 7 days of index ICU discharge (late event group; n = 129, 19%).

**Fig 2:**
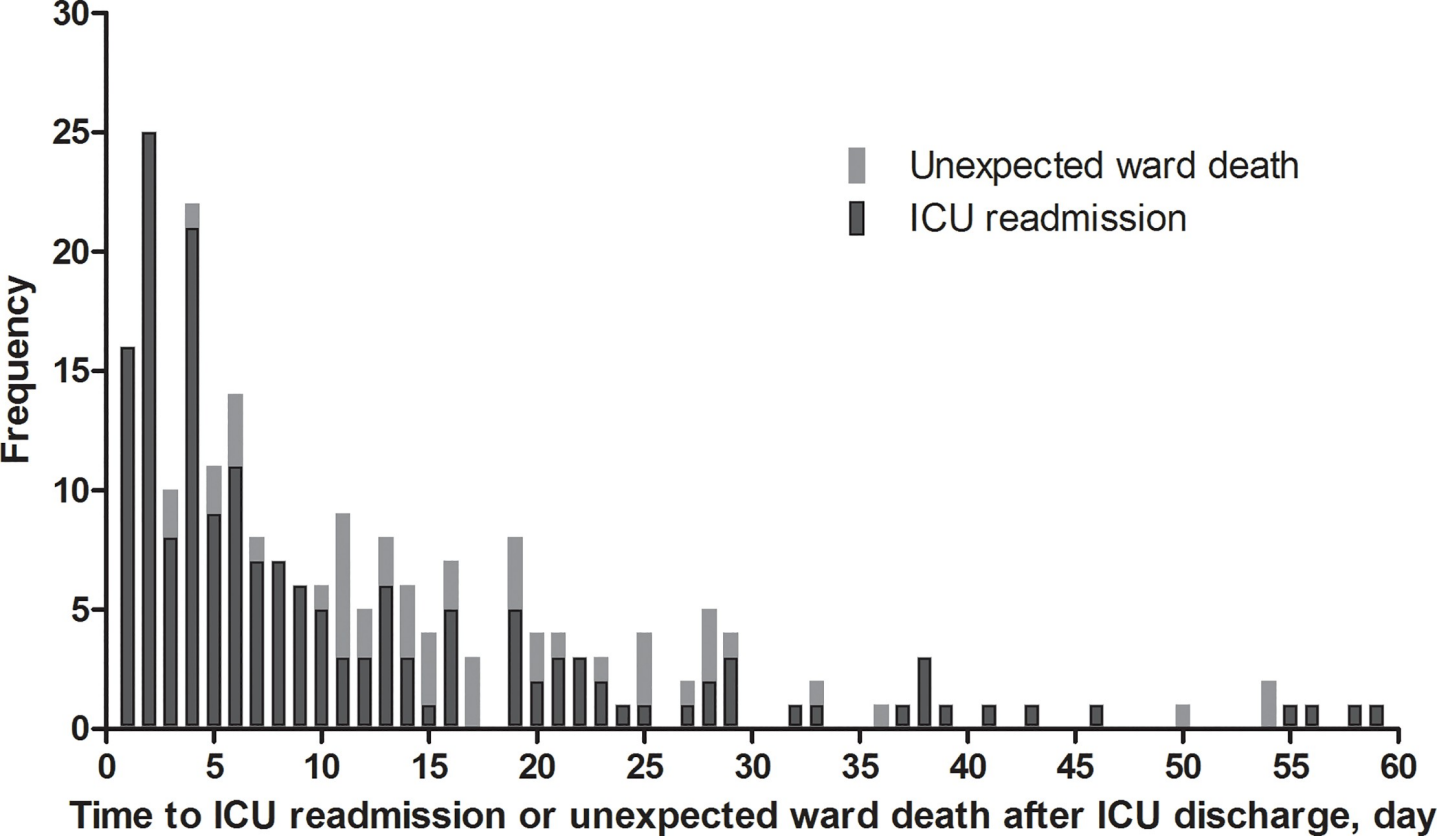
Intensive care unit (ICU) readmission or unexpected death on the ward after being discharged alive from the ICU.

The number of ICU readmissions and unexpected ward death after index ICU discharge is shown in Fig 2.

### Baseline characteristics

Baseline characteristics and clinical course of index ICU admission are shown in Table 1. There were 243 (35.2%) patients with hematologic malignancy, and this was more common in the early (50.0%), intermediate (45.3%), and late event groups (44.2%) than in the no event group (29.8%, *P* = 0.001). In the late event group, respiratory failure as the reason for index ICU admission was more common (39.5% *vs*. 25.4%, *P* = 0.010), whereas cardiovascular problems were less frequent (11.6% *vs*. 25.7%, *P* = 0.001) than in the no event group. SAPS 3 and SOFA scores were higher in the early and late event groups than in the no event group. Mechanical ventilation during index ICU admission was more frequently required in the early (61.9%), intermediate (46.9%), and late event groups (45.7%) than in the no event group (30.9%, *P* < 0.001). Finally, the LOS of index ICU admission was longer in the three event groups than in the no event group (*P <* 0.001). Although SOFA scores at index ICU discharge were higher in the three event groups than in the no event group (*P* < 0.001), there were no significant differences in the difference values of SOFA between admission and discharge across the four groups (*P* = 0.540).

**Table 1:** Baseline characteristics and clinical course of index ICU admission.

Variables			Total (N = 691)	No event group* (n = 456)	Early event group* (n = 42)	Intermediate event group* (n = 64)	Late event group* (n = 129)	*P*
Age, yr			62 (51–70)	63 (51–70)	62 (51–72)	63 (53–68)	62 (50–72)	0.997
Sex, male			456 (66.0)	300 (65.8)	27 (64.3)	52 (81.3)	77 (59.7)	0.029^b^
ECOG ≥ 3			97 (14.0)	47 (10.3)	11 (26.2)	11 (17.2)	28 (21.7)	0.001^a^^,^^c^
Body mass index, kg/m^2^			21.4 (19.3–23.7)	21.6 (19.4–24.0)	20.5 (18.9–23.8)	22.1 (20.1–24.0)	20.8 (18.7–23.0)	0.104
Comorbidity								
	Diabetes mellitus		157 (22.7)	98 (21.5)	11 (26.2)	16 (25.0)	32 (24.8)	0.756
	Chronic heart disease		82 (11.9)	48 (10.5)	6 (14.3)	3 (4.7)	25 (19.4)	0.012^c^
	Chronic liver disease		62 (9.0)	46 (10.1)	2 (4.8)	2 (3.1)	12 (9.3)	0.248
	Cerebrovascular disease		54 (7.8)	31 (6.8)	4 (9.5)	6 (9.4)	13 (10.1)	0.486
	Chronic kidney disease		52 (7.5)	31 (6.8)	5 (11.9)	5 (7.8)	11 (8.5)	0.550
	COPD/asthma		34 (4.9)	16 (3.5)	4 (9.5)	4 (6.3)	10 (7.8)	0.063
Status of malignancy								
	Oncologic malignancy		448 (64.8)	320 (70.2)	21 (50.0)	35 (54.7)	72 (55.8)	0.001^a^^,^^b^^,^^c^
		Extensive disease	277/448 (61.8)	192/320 (60.0)	15/21 (71.4)	23/35 (65.7)	47/72 (65.3)	0.602
	Hematologic malignancy		243 (35.2)	136 (29.8)	21 (50.0)	29 (45.3)	57 (44.2)	0.001^a^^,^^b^^,^^c^
		Stem cell transplantation	54/243 (22.2)	28/136 (20.6)	6/21 (28.6)	5/29 (17.2)	15/57 (26.3)	0.629
	First presentation of malignancy		142 (20.5)	88 (19.3)	9 (21.4)	18 (28.1)	27 (20.9)	0.437
	Chemotherapy within 4 weeks		319 (46.2)	218 (47.8)	22 (52.4)	31 (48.4)	48 (37.2)	0.142
Source of index ICU admission								<0.001^a^^b^^,^^c^
	Emergency department		338 (48.9)	256 (56.1)	19 (45.2)	22 (34.4)	41 (31.8)	
	Others^†^		353 (51.1)	200 (43.9)	23 (54.8)	42 (65.6)	88 (68.2)	
Reason for index ICU admission								
	Sepsis		240 (34.7)	150 (32.9)	19 (45.2)	19 (29.7)	52 (40.3)	0.157
	Respiratory failure		201 (29.1)	116 (25.4)	11 (26.2)	23 (35.9)	51 (39.5)	0.010^c^
	Cardiovascular		147 (21.3)	117 (25.7)	7 (16.7)	8 (12.5)	15 (11.6)	0.001^c^
	Bleeding		43 (6.2)	31 (6.8)	1 (2.4)	6 (9.4)	5 (3.9)	0.343
	Neurologic disorder		19 (2.7)	12 (2.6)	1 (2.4)	3 (4.7)	3 (2.3)	0.733
	Others		41 (5.9)	30 (6.6)	3 (7.1)	5 (7.8)	3 (2.3)	0.202
SAPS III at index ICU admission			49 (40–60)	47 (37–56)	61 (48–71)	53 (40–66)	54 (44–64)	<0.001^a^^,^^c^
SOFA score at index ICU admission			5 (3–8)	5 (2–8)	8 (6–10)	6 (3–9)	6 (4–9)	<0.001^a^^,^^c^
Organ support during index ICU admission								
	Vasopressor		310 (44.9)	193 (42.3)	25 (59.5)	26 (40.6)	66 (51.2)	0.061
	Mechanical ventilator		256 (37.0)	141 (30.9)	26 (61.9)	30 (46.9)	59 (45.7)	<0.001^a^^,^^b^^,^^c^
	Renal replacement therapy		64 (9.3)	35 (7.7)	5 (11.9)	8 (12.5)	16 (12.4)	0.203
LOS of index ICU admission			3 (2–6)	3 (2–5)	5 (2–10)	4 (2–8)	4 (2–8)	<0.001^a^^,^^b^^,^^c^
SOFA at index ICU discharge			3 (1–6)	3 (1–5)	5 (4–7)	4 (1–6)	4 (2–6)	<0.001^a^^,^^c^
Difference value of SOFA between admission and discharge			-2 (-4 to 0)	-1 (-3 to 0)	-2 (-4 to 0)	-2 (-4 to 0)	-2 (-4 to -1)	0.540

Values are median with interquartile range or n (%).

*No event group means patients who were discharged alive from the hospital without ICU readmission. Early event group means patients who were unexpectedly readmitted to the ICU or died at the ward within 2 days after index ICU discharge. Intermediate event group means patients who were unexpectedly readmitted to the ICU or died at the ward between 2 and 7 days after index ICU discharge. Late event group means patients who were unexpectedly readmitted to the ICU or died at the ward after 7 days of index ICU discharge.

^a^*P* < 0.05 with Bonferroni correction between no event and early event groups.

^b^*P* < 0.05 with Bonferroni correction between no event and intermediate event groups.

^c^*P* < 0.05 with Bonferroni correction between no event and late event groups.

^†^Transfer from a ward or outside hospital.

ECOG, Eastern Cooperative Oncology Group; COPD, chronic obstructive pulmonary disease; LOS, length of stay; ICU, intensive care unit; SAPS, Simplified Acute Physiology Score; SOFA, Sequential Organ Failure Assessment.

### Index ICU discharge date

Vital signs and laboratory findings on the day of index ICU discharge are shown in Table 2. Heart rate was higher in the early (median 101/min), intermediate (median 99/min), and late event groups (median 97/min) than in the no event group (median 91/min; *P* < 0.001). Patients with a lower GCS were more commonly identified in the early event group than in the no event group (*P* = 0.001); however, there were no differences between the other event groups and the no event group. Of 657 patients that underwent arterial blood gas analyses, there were no significant differences across the groups. There were statistically significant differences in the laboratory findings of platelet counts, total bilirubin, albumin, urea nitrogen, lactic acid, and N-terminal pro-brain natriuretic peptide (NT-proBNP); however, there were missing values for some variables.

**Table 2:** Vital signs, Glasgow coma scale, and laboratory findings on the day of index ICU discharge.

Variables		Total (N = 691)	No event group* (n = 456)	Early event group* (n = 42)	Intermediate event group* (n = 64)	Late event group* (n = 129)	*P*
Vital sign							
	Mean ABP, mmHg	87 (76–97)	83 (75–95)	91 (80–100)	92 (83–103)	89 (77–97)	0.001^b^
	Heart rate, /min	92 (79–105)	91 (78–102)	101 (88–118)	99 (83–112)	97 (83–108)	<0.001^a^^,^^b^^,^^c^
	Respiratory rate, /min	20 (16–24)	19 (16–23)	22 (17–27)	21 (16–24)	21 (17–25)	0.047
	Body temperature, °C	36.6 (36.3–37.1)	36.6 (36.3–37.1)	36.8 (36.5–37.2)	36.7 (36.3–37.2)	36.6 (36.2–36.9)	0.161
Glasgow coma scale							0.001^a^
	15	515 (74.5)	350 (76.8)	19 (45.2)	47 (73.4)	99 (76.7)	
	11–14	143 (20.7)	87 (19.1)	16 (38.1)	15 (23.4)	25 (19.4)	
	≤10	33 (4.8)	19 (4.2)	7 (16.7)	2 (3.1)	5 (3.9)	
Arterial blood gas (n = 657)							
	PaO_2_/FiO_2_	405 (355–479)	403 (356–480)	419 (345–510)	407 (357–443)	418 (352–507)	0.819
	pH	7.47 (7.44–7.50)	7.47 (7.44–7.50)	7.46 (7.41–7.50)	7.48 (7.44–7.50)	7.47 (7.44–7.50)	0.671
	PaCO_2_, mmHg	33.7 (30.4–38.5)	33.5 (30.2–37.9)	35.6 (32.2–40.8)	33.8 (31.7–38.8)	33.8 (30.0–39.8)	0.058
	HCO_3_, mmol/L	24.9 (22.0–28.1)	24.5 (21.8–27.9)	26.0 (24.0–29.4)	25.5 (23.1–28.7)	24.9 (21.5–28.3)	0.063
Laboratory finding							
	White blood cell, ×10^3^/μL	7.26 (4.16–11.55)	7.21 (4.45–11.14)	7.07 (1.96–12.95)	7.46 (3.39–12.53)	7.75 (2.89–13.13)	0.902
	Hemoglobin, g/dL	9.9 (9.0–11.2)	10.0 (9.2–11.2)	9.6 (8.9–10.3)	9.7 (9.0–11.0)	9.8 (8.9–11.4)	0.067
	Platelets, ×10^3^/μL	107 (40–214)	130 (48–229)	51 (22–92)	63 (32–175)	77 (35–175)	<0.001^a^^,^^b^^,^^c^
	Total bilirubin, mg/dL	0.8 (0.5–1.5)	0.7 (0.4–1.5)	1.1 (0.6–2.9)	1.0 (0.6–1.8)	0.8 (0.5–1.5)	0.002^a^^,^^b^
	Albumin, g/dL (n = 620)	2.9 (2.6–3.2)	2.9 (2.6–3.3)	2.8 (2.5–3.0)	2.8 (2.5–3.1)	2.8 (2.5–3.1)	0.036
	Urea nitrogen, mg/dL	20.4 (13.0–30.2)	17.9 (12.6–27.2)	24.1 (12.1–34.0)	27.3 (17.7–40.2)	22.7 (15.7–33.2)	<0.001^b^^,^^c^
	Creatinine, mg/dL	0.7 (0.5–1.0)	0.7 (0.5–1.0)	0.6 (0.5–1.0)	0.8 (0.5–1.2)	0.7 (0.5–1.2)	0.657
	Sodium, mmol/L (n = 687)	138 (134–141)	138 (134–141)	139 (135–143)	137 (135–142)	137 (134–140)	0.282
	Lactic acid, mmol/L (n = 627)	1.39 (1.02–1.97)	1.34 (1.00–1.87)	1.67 (1.31–2.51)	1.58 (0.99–2.22)	1.39 (1.04–2.13)	0.003^a^
	CRP, mg/dL (n = 545)	6.6 (3.2–12.8)	6.6 (3.1–13.0)	6.9 (2.6–12.1)	7.1 (2.3–15.2)	6.1 (3.8–11.3)	0.955
	Procalcitonin, ng/mL (n = 402)	0.94 (0.23–5.86)	0.96 (0.22–6.37)	1.57 (0.40–7.73)	0.82 (0.22–2.97)	0.78 (0.26–4.61)	0.692
	NT-proBNP, pg/mL (n = 332)	1379 (508–5676)	1167 (458–5077)	4233 (1336–17252)	1927 (263–4930)	1620 (699–7488)	0.007^a^

Values are median with interquartile range or n (%).

*No event group means patients who were discharged alive from the hospital without ICU readmission. Early event group means patients who were unexpectedly readmitted to the ICU or died at the ward within 2 days after index ICU discharge. Intermediate event group means patients who were unexpectedly readmitted to the ICU or died at the ward between 2 and 7 days after index ICU discharge. Late event group means patients who were unexpectedly readmitted to the ICU or died at the ward after 7 days of index ICU discharge.

^a^*P* < 0.05 with Bonferroni correction between no event and early event groups.

^b^*P* < 0.05 with Bonferroni correction between no event and intermediate event groups.

^c^*P* < 0.05 with Bonferroni correction between no event and late event groups.

ICU, intensive care unit; ABP, arterial blood pressor; CRP, C-reactive protein; NT-proBNP, N-terminal pro-brain natriuretic peptide.

### ICU readmission or unexpected ward death and final outcomes

Patient characteristics of the event groups are shown in Table 3. Unexpected ward death was more frequent in the intermediate (12.5%) and late event groups (37.2%) than in the early event group (2.4%, *P* < 0.001). There were no differences in reason for ICU readmission across the event groups. Of 178 patients readmitted to the ICU, readmissions for the same reason as index admissions were found in 98 (55.1%) cases. There were no differences on SAPS 3 and SOFA scores at ICU readmission across the event groups. In addition, there was no difference in the mortality of ICU readmission across the event groups (early *vs*. intermediate *vs*. late; 26.8% *vs*. 32.1% *vs*. 30.9%; *P* = 0.845). However, hospital mortality was higher in the late event group than in the early event group (early *vs*. intermediate *vs*. late; 54.8% *vs*. 65.6% *vs*. 76.0%; *P* = 0.026).

**Table 3:** ICU readmission status and final outcomes.

Variables		Total (N = 235)	Early event group* (n = 42)	Intermediate event group* (n = 64)	Late event group* (n = 129)	*P*
Death at general ward before ICU readmission		57 (24.3)	1 (2.4)	8 (12.5)	48 (37.2)	<0.001^a^^,^^b^^,^^c^
Reason for ICU readmission						
	Respiratory failure	74/178 (41.6)	18/41 (43.9)	24/56 (42.9)	32/81 (39.5)	0.873
	Sepsis	60/178 (33.7)	12/41 (29.3)	15/56 (26.8)	33/81 (40.7)	0.187
	Cardiovascular	12/178 (6.7)	4/41 (9.8)	4/56 (7.1)	4/81 (4.9)	0.590
	Bleeding	9/178 (5.1)	2/41 (4.9)	4/56 (7.1)	3/81 (3.7)	0.685
	Neurologic disorder	9/178 (5.1)	2/41 (4.9)	5/56 (8.9)	2/81 (2.5)	0.273
	Others	14/178 (7.9)	3/41 (7.3)	4/56 (7.1)	7/81 (8.6)	1.000
	Same reason as index ICU	98/178 (55.1)	22/41 (53.7)	34/56 (60.7)	42/81 (51.9)	0.579
SAPS III at ICU readmission		64 (50–75)	66 (48–76)	58 (44–78)	65 (53–72)	0.497
SOFA at ICU readmission		8 (5–12)	9 (5–12)	7 (5–12)	8 (5–11)	0.615
Mortality of ICU readmission		54/178 (30.3)	11/41 (26.8)	18/56 (32.1)	25/81 (30.9)	0.845
LOS of ICU readmission		4 (2–9)	7 (4–10)	5 (2–11)	3 (1–8)	0.017^b^
Hospital LOS ^d^		39 (24–77)	36 (20–66)	34 (21–63)	49 (28–84)	0.015^c^
Hospital mortality ^e^		163 (69.4)	23 (54.8)	42 (65.6)	98 (76.0)	0.026^c^

Values are median with interquartile range or n (%).

*Early event group means patients who were unexpectedly readmitted to the ICU or died at the ward within 2 days after index ICU discharge. Intermediate event group means patients who were unexpectedly readmitted to the ICU or died at the ward between 2 and 7 days after index ICU discharge. Late event group means patients who were unexpectedly readmitted to the ICU or died at the ward after 7 days of index ICU discharge.

^a^*P* < 0.05 with Bonferroni correction between early and intermediate event groups.

^b^*P* < 0.05 with Bonferroni correction between early and late event groups.

^c^*P* < 0.05 with Bonferroni correction between intermediate and late event groups.

^d^Hospital LOS in no event group was a median of 17 days (IQR, 9–30 days).

^e^Because the no event group was defined as patients who were discharged alive from the hospital without ICU readmission, there were no hospital mortalities in the no event group. Of a total of 691 patients, the hospital mortality was 163 (23.6%).

ICU, intensive care unit; SAPS, Simplified Acute Physiology Score; SOFA, Sequential Organ Failure Assessment; LOS, length of stay; IQR, interquartile range.

### Independent variables associated with ICU readmission or unexpected ward death

Multinomial logistic regression analysis to identify the independent variables associated with unplanned ICU readmission or unexpected ward death depending on the time and the index ICU discharge is shown in Table 4. Comorbidities such as chronic obstructive pulmonary disease (COPD) and asthma, mechanical ventilation during index ICU admission, lower GCS, and platelet count on the day of index ICU discharge were associated with early event group. Index ICU admission from the ER and higher urea nitrogen on the day of index ICU discharge were more frequent in the intermediate event group. The late event group was associated with comorbidity such as chronic heart disease and COPD/asthma, chemotherapy within 4 weeks before index ICU admission, index ICU admission from the ER, reasons for index ICU admission including sepsis and respiratory failure, a heart rate more than 100 beats/min, and higher blood urea nitrogen on the day of index ICU discharge. While different variables were associated with ICU readmission or ward death, none of the associated variables were statistically significant across the three event groups.

**Table 4:** Multinomial logistic regression analysis of ICU readmission or ward death after ICU discharge^†^.

Variables			к^2^	*P*	Early event group*		Intermediate event group*		Late event group*	
					aOR (95% CI)	*P*	aOR (95% CI)	*P*	aOR (95% CI)	*P*
Sex, male			8.94	0.030	0.83 (0.40–1.73)	0.622	1.90 (0.96–3.76)	0.066	0.66 (0.42–1.02)	0.064
Comorbidity										
	Chronic heart disease		11.60	0.009	1.14 (0.41–3.12)	0.803	0.36 (0.11–1.24)	0.105	2.12 (1.17–3.83)	0.013
	Chronic liver disease		7.81	0.050	0.24 (0.05–1.19)	0.081	0.24 (0.05–1.08)	0.062	0.81 (0.38–1.74)	0.587
	COPD/asthma		10.72	0.013	9.32 (2.45–35.45)	0.001	1.98 (0.59–6.69)	0.271	2.52 (1.02–6.28)	0.046
Chemotherapy within 4 weeks			8.04	0.045	0.68 (0.32–1.42)	0.304	0.86 (0.48–1.55)	0.617	0.52 (0.33–0.83)	0.006
Source of index ICU admission: ER			15.97	0.001	1.30 (0.64–2.60)	0.468	2.05 (1.15–3.67)	0.015	2.20 (1.41–3.42)	<0.001
Reason for index ICU admission										
	Sepsis		8.77	0.033	1.27 (0.52–3.12)	0.598	0.68 (0.32–1.45)	0.315	2.15 (1.18–3.91)	0.012
	Respiratory failure		11.05	0.011	0.68 (0.26–1.84)	0.451	1.01 (0.48–2.11)	0.981	2.52 (1.38–4.63)	0.003
Organ support during index ICU admission: mechanical ventilation			10.91	0.012	3.30 (1.55–7.00)	0.002	1.52 (0.82–2.83)	0.187	1.32 (0.82–2.13)	0.251
On the day of index ICU discharge										
	Heart rate > 100/min		15.77	0.001	2.63 (1.31–5.26)	0.007	1.73 (0.98–3.06)	0.060	2.02 (1.30–3.14)	0.002
	GCS									
		15	18.12	0.006	1.00 (reference)		1.00 (reference)		1.00 (reference)	
		11–14			2.64 (1.22–5.73)	0.014	0.96 (0.48–1.91)	0.908	0.69 (0.40–1.20)	0.186
		≤ 10			6.92 (2.22–21.55)	0.001	0.54 (0.11–2.63)	0.446	0.74 (0.25–2.18)	0.589
	Platelet									
		>100, ×10^3^/μL	19.48	0.003	1.00 (reference)		1.00 (reference)		1.00 (reference)	
		>50 but ≤100, ×10^3^/μL			5.63 (1.96–16.14)	0.001	0.93 (0.36–2.41)	0.875	1.73 (0.93–3.22)	0.081
		≤50, ×10^3^/μL			5.95 (2.19–16.19)	<0.001	1.68 (0.83–3.42)	0.150	1.49 (0.86–2.58)	0.155
	Urea nitrogen									
		≤20 mg/dL	18.42	0.005	1.00 (reference)		1.00 (reference)		1.00 (reference)	
		>20 but ≤40 mg/dL			0.72 (0.32–1.60)	0.413	2.33 (1.21–4.45)	0.011	1.82 (1.13–2.93)	0.015
		>40 mg/dL			0.65 (0.24–1.77)	0.398	3.51 (1.55–7.97)	0.003	1.88 (0.99–3.58)	0.056

^†^ No event group was used as the reference category.

*No event group means patients who were discharged alive from the hospital without ICU readmission. Early event group means patients who were unexpectedly readmitted to the ICU or died at the ward within 2 days after index ICU discharge. Intermediate event group means patients who were unexpectedly readmitted to the ICU or died at the ward between 2 and 7 days after index ICU discharge. Late event group means patients who were unexpectedly readmitted to the ICU or died at the ward after 7 days of index ICU discharge.

This multinomial logistic regression analysis model had the following Pearson goodness-of-fit test result: χ^2^ = 2036.173 (*P* = 0.408). ICU, intensive care unit; aOR, adjusted odds ratio; CI, confidence interval; COPD, chronic obstructive pulmonary disease; ER, emergency room; GCS, Glasgow coma scale.

## Discussion

In this study, we investigated the epidemiology, characteristics including disease-specific and treatment-related variables, and clinical outcomes of patients who experienced ICU readmission or death in the ward based on the time interval between discharge and readmission or unexpected ward death and compared them with those of patients who were discharged from the hospital alive without ICU readmission during their same hospitalization.

Overall, 34.0% of patients in our study were readmitted to the ICU (25.8%) or died in the general ward (8.2%) during hospitalization, and 6.1%, 9.2%, and 18.7% of readmission or death in the ward occurred within 2 days, 2–7 days, and 7 days after ICU discharge, respectively. Sepsis and respiratory failure were common causes and accounted for 60–80% of ICU admission, regardless of whether it was the index admission or readmission and when the event occurred. Recent studies of the general population demonstrated that 3–17% of patients discharged from a mixed medical-surgical or medical ICU were readmitted to the ICU during the same hospitalization [15,19,20]. Although there is currently limited data on critically ill cancer patients, ICU readmission rates in this population are reported to be about 8–9% [21,22]. Readmission to the ICU occurred at a relatively high rate in our study compared to the incidence reported in previous studies. This readmission rate could have been impacted by several factors. Although we excluded patients who decided not to be readmitted to the ICU, the severity of illness of our cohort was much higher than in previous studies [14,15,21,23]. The severity of illness at the time of ICU admission, which is assessed by various scoring systems, is known to be an independent risk factor for ICU readmission [14,19,24]. In addition, cancer is a comorbidity that can increase readmission rates [19,25].

Various patient-related factors such as age, male gender, comorbid conditions, admission diagnosis, use of organ support treatment during ICU stay, and ICU LOS [5,14,16,17,21,23,26] and organizational factors such as lack of ICU beds and out-of-hours discharge from the ICU have been identified as variables associated with increased ICU readmission or unexpected ward death in previous studies [17,18,23,27]. However, previous reports had various criteria for the timing of ICU readmission (within 24 hours [18], within 48 hours [14,16], within 7 days [17], and no limitation [5,21,23,27]). In addition, several reports excluded unexpected death on the ward in the analysis [5,21,23,27]. Although ICU readmission and unexpected death on the ward might have common risk factors, it might vary depending on the timing of ICU readmission and death on the ward after index ICU discharge. Therefore, our study identified variables that were related to readmission to the ICU or death on the ward within 2 days, between 2 and 7 days, and after 7 days following index ICU discharge. Organ dysfunction on the day of index ICU discharge (decreased mental status, tachycardia, and thrombocytopenia) and organ support treatment during index ICU stay (need for mechanical ventilation) mainly affected the early readmitted or unexpectedly died patients. Considering that the median SOFA score at the index ICU discharge in the early event group was higher than in other groups, ICU discharge before stabilization of acute illness could be related to early readmission or death on the ward. This is supported by the European Society of Intensive Care Medicine, indicating that the ICU readmission rate within 48 hours of ICU discharge is mainly explained by residual organ dysfunction/failure at ICU discharge [28].

Although specific reasons for index ICU admission including sepsis and respiratory failure was associated with late readmission or unexpected ward death, we hypothesized that the patient’s medical condition after sepsis or respiratory failure, such as worsening pre-illness status, new impaired function, and frailty, might affect readmission or death more than incomplete recovery from acute illness [29,30]. As a result, an underlying medical condition such as chronic heart disease, COPD/asthma, and chemotherapy within 4 weeks influenced ICU readmission or death on the ward after 7 days of ICU discharge. Therefore, improving general conditions and managing chronic illness might be an effective way to prevent late readmission or unexpected death on the ward.

Previous studies demonstrated that patients with at least one ICU readmission had a higher mortality than patients who did not [19,21]. In our cohort, mortality rate at index ICU hospitalization was 23.6%, and overall ICU mortality of patients readmitted to the ICU was 30.3%. ICU mortalities among patients readmitted to the ICU within 2 days, between 2 and 7 days, and after 7 days following index ICU discharge were similar, but hospital mortality in the late event group was higher than others, and patients typically had a longer hospital stay. This is in agreement with previous studies showing that ICU readmission is associated with a longer hospital stay [4,5].

This study provides information on factors associated with ICU readmission or unexpected ward death and clinical outcomes in critically ill cancer patients discharged alive from the ICU. Furthermore, we characterized cancer patients who were readmitted to the ICU or unexpectedly died on the ward based on the time interval between ICU discharge and the events as early, intermediate, and late. However, there were several limitations that should be considered. First, given the retrospective and observational nature of our study, there is a potential risk of selection bias and confounding variables influencing the significance of our findings. However, the data were prospectively collected from all of the critically ill cancer patients consecutively admitted to the medical ICU. In addition, our study was conducted at a single institution with a specialized ICU for critically ill cancer patients. Considering that rates of readmission to the ICU varied by geographic region, hospital, and type of ICU [31,32], the generalizability of our findings to other centers is limited. Second, some of the associated factors to the early events such as tachycardia and mental deterioration could indicate a premature ICU discharge, since there was no predefined objective criterion for ICU discharge. Third, we used the time interval from ICU discharge to ICU transfer in order to divide the group of patients readmitted to the ICU or unexpectedly died on the ward. Therefore, it is difficult to exclude the possibility that there is a gap between the time of decision to readmit to the ICU and the time transferred to the ICU due to delayed transfers due to lack of bed availability. Fourth, the information about limitation of care decision, which is a variable that may affect the mortality, collected only at the time of the discharge from ICU and changes in the decision were not followed during the rest of the hospitalization. In addition, we only focused on the patient-related factors in the analysis, without consideration of organizational factors relevant to readmission according to previous studies [18,33].

## Conclusions

In summary, about one-third of critically ill cancer patients were readmitted to the ICU or died on the ward after being discharged alive from the index ICU admission. Risk factors associated with ICU readmission and mortality were different across the time intervals between discharge and events. Therefore, it is necessary to carefully assess residual organ dysfunction at ICU discharge as well as the treatment of underlying disease after ICU discharge in order to reduce ICU readmission and hospital mortality in critically ill cancer patients.
