# Correction: Readmission and hospital mortality after ICU discharge of critically ill cancer patients

**DOI:** 10.1371/journal.pone.0218196

**Published:** 2019-06-05

**Authors:** Byeong-Ho Jeong, Soo Jin Na, Dae-Sang Lee, Chi Ryang Chung, Gee Young Suh, Kyeongman Jeon

The Data Availability statement is incorrect. The raw data underlying the study are not provided in the published paper and its Supporting Information files. The authors have provided the data as Supporting Information file S1 File.

## Supporting information

S1 File De-identified data set. (CSV)
